# Outcomes of oblique lateral interbody fusion for degenerative lumbar disease in patients under or over 65 years of age

**DOI:** 10.1186/s13018-018-0740-2

**Published:** 2018-02-20

**Authors:** Chengzhen Jin, Milin S. Jaiswal, Sin-Soo Jeun, Kyeong-Sik Ryu, Jung-Woo Hur, Jin-Sung Kim

**Affiliations:** Spine Center, Department of Neurosurgery, Seoul St. Mary’s Hospital, College of Medicine, The Catholic University of Korea, Seoul, South Korea, 222 Banpo-daero Seocho-gu, Seoul, 06591 Republic of Korea

**Keywords:** Oblique lateral interbody fusion, Co-morbidity, Complication, Elderly patients, Degenerative

## Abstract

**Background:**

Oblique lateral interbody fusion (OLIF) offers the solution to problems of anterior lumbar interbody fusion (ALIF) and lateral lumbar interbody fusion (LLIF). However, OLIF technique for degenerative spinal diseases of elderly patients has been rarely reported. The objective of this study was to determine the clinical and radiological results of OLIF technique for degenerative spinal diseases in patients under or over 65 years of age.

**Methods:**

Sixty-three patients who underwent OLIF procedure were enrolled, including 29 patients who were less than 65 years of age and 34 patients who were over 65 years of age. Fusion rate, change of disc height and lumbar lordotic angle, Numeric Rating Scale (NRS), return to daily activity, patient’s satisfaction rate (PSR), and Oswestry disability index (ODI) were used to assess clinical and functional outcomes.

**Results:**

The mean NRS scores for back and leg pain decreased, respectively, from 4.6 and 5.9 to 2.3 and 1.8 in the group A (less than 65 years) and from 4.5 and 6.8 to 2.6 and 2.2 in the group B (over 65 years) at the final follow-up period. The mean ODI scores improved from 48.4 to 24.0% in the group A and from 46.5 to 25.2% in the group B at the final follow-up period. In both groups, the NRS and ODI scores significantly changed preoperatively to postoperatively (*p* <  0.001). However, statistical analysis yielded no significant difference in postoperative NRS/ODI scores between two groups. In both groups, the changes in the disc height, segmental lordosis, and fusion rate between the preoperative and postoperative periods were significant. The amount of change between preoperative and postoperative disc height, segmental lordosis, and whole lumbar lordosis demonstrated significant intergroup differences (*p* <  0.05). Overall perioperative complications occurred in 8 of 29 (27.6%) patients in the group A and in 10 of 34 (29.4%) patients in the group B. In both groups, the major complication incidence was 0 and 3%, respectively.

**Conclusion:**

Although there was the slightly high incidence of complication associated with high rate of co-morbidities in elderly patients, OLIF for degenerative lumbar diseases in elderly patients showed favorable clinical and radiological outcomes.

## Background

For years, lumbar interbody fusion has been used as a powerful surgical tool for various pathologies of the lumbar spine including degenerative disc disease (DDD), spondylolisthesis, disc herniation, and deformity [1, 2]. Conventional anterior lumbar interbody fusion (ALIF) [3] and posterior/transforaminal lumbar interbody fusion (PLIF/TLIF) [4–6] techniques have given excellent fusion and clinical results. However, they have their own restrictions and disadvantages. Surgical approaches have also been improved with new innovations including the introduction of lateral lumbar interbody fusion (LLIF) [7–9], a lateral approach between the retroperitoneal space and psoas muscle to allow access to the lumbar spine. To decrease surgical trauma, reduce operative bleeding and infection incidence, and reduce hospital stay, minimally invasive spinal (MIS) surgical approaches and techniques are more and more refined since their first introduction [10]. To counter approach-related hurdles of ALIF and LLIF, oblique lateral interbody fusion (OLIF) has been proposed as a solution to access lumbar disc space by taking advantages of the surgical space between the aorta and psoas muscle [11, 12]. With better understanding and refinement of MIS surgical techniques, tides are turning from traditional PLIF/TLIF to MIS-TLIF and traditional ALIF/LLIF to OLIF.

Although clinical outcomes and complications of various lumbar interbody fusions have been assessed in many studies [13, 14], OLIF technique for the degenerative spinal disease of elderly patients has been rarely reported. Therefore, the objective of this study was to determine the clinical and radiological results of OLIF technique in patients under and over 65 years of age and compare the differences between the two groups.

## Methods

### Patient population

From June 2013 to May 2016, 86 patients who underwent OLIF procedure for the degenerative spinal disease were enrolled in this study. OLIF was performed by a single surgeon. Among these 86 patients, patients who had neoplasm, infectious disease, trauma, more than three-level fusion surgeries, deformity of more than 20° on coronal plane, or follow-up period of fewer than 1 year were excluded. Patients who suffered from low back pain and/or leg pain with neurogenic intermittent claudication and progressive neurological deficit and confirmed to have lumbar stenosis, spondylolisthesis, and herniated nucleus pulposus (HNP) by radiological examinations were included. Before surgical intervention was advised, conservative treatment for over 2 months failed in these patients. After excluding 23 patients who met the exclusion criteria, a total of 63 patients who met the inclusion criteria were finally included in this study, including 29 (46.03%) patients who were less than 65 years of age (group A) and 34 (53.97%) patients who were over 65 years of age (group B). Of the 63 patients, 43 (68.3%) had no previous history of lumbar surgery, 11 (17.4%) had adjacent segment disease (ASD) with the previous history of lumbar fusion surgery, and 9 (14.3%) had previous posterior lumbar decompression without stabilization surgery. Medical records of these 63 patients were reviewed to obtain demographic data, primary diagnosis, and the length of hospital stay, preoperative co-morbidities, segmental fusion status, and postoperative complications. This study was approved by institutional review board (IRB) of our institution. Informed written consents were obtained from all the subjects participating in the study.

### Operative technique

**Fig. 1:**
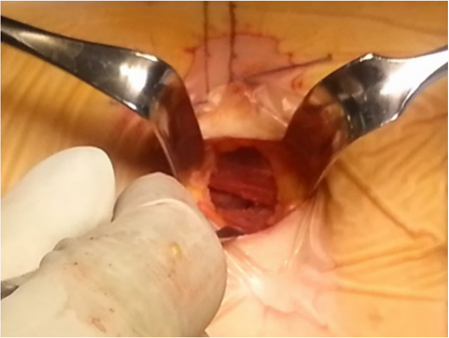
A 2.5–3.0-cm skin incision was made centered in projection of the target segment and parallel to external oblique muscle fibers

**Fig. 2:**
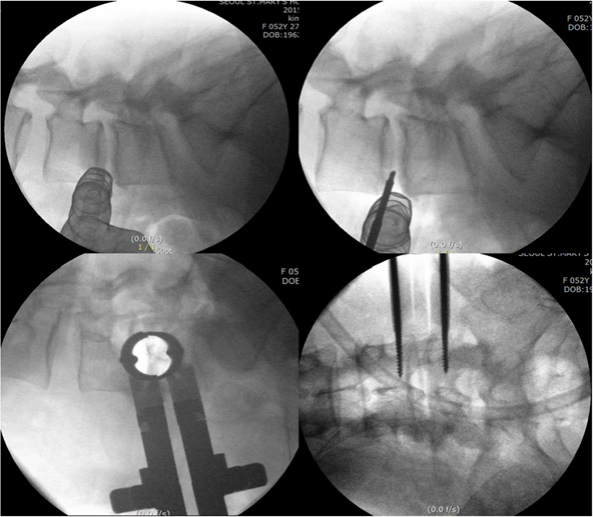
The psoas muscle was dissected with the index finger, and tubular retractor system was docked in the targeting disc level

**Fig. 3:**
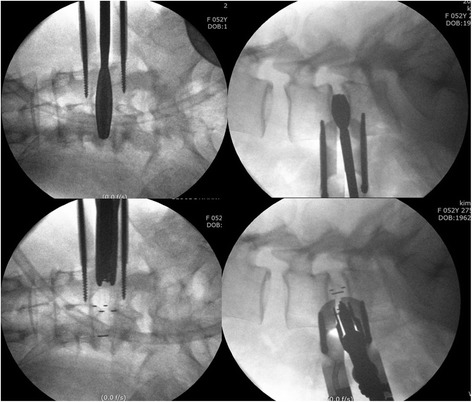
Vertebral endplates were prepared, and cage packed with graft bone was inserted to the disc space

**Fig. 4:**
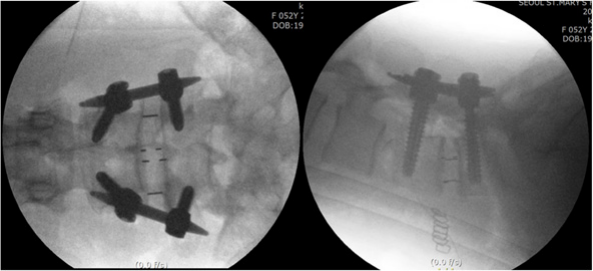
Posterior pedicle screw fixation was performed using percutaneous approach

Patient was placed in a right-sided lateral decubitus position. Under fluoroscopic control, anatomical surface of the disc in true lateral view was marked on the skin. Standard preoperative preparation of surgical field was done. For single-level fusion, 2.5–3.0-cm skin incision was made centered in the projection of the target segment and parallel to external oblique muscle fibers (Fig. 1). External oblique muscle, internal oblique muscle, and transverse abdominal muscle were dissected along the direction of their fibers with a blunt muscle-splitting technique. Retroperitoneal space was accessed by blunt dissection along the retroperitoneal fat tissue. The peritoneal sac was mobilized anteriorly. The psoas muscle was dissected with the index finger and retracted posteriorly. The targeted disc space was exposed and tubular retractor system was docked (Fig. 2). Special attention was given to the genitofemoral nerve, the sympathetic chain, and segmental blood vessels. In multi-level cases, the incision was enlarged up to 4.0–6.0 cm or multiple skin incisions of 2.5–3.0 cm per level were made. Imaging guidance (fluoroscopy) was used to confirm the correct level. After discectomy, vertebral endplates were prepared and the subchondral bone was exposed. To achieve interbody fusion, cage packed with demineralized bone matrix (DBM) or harvests of iliac bone was inserted (Fig. 3). After the anterolateral procedure, posterior lumbar stabilization was performed with percutaneous pedicle screw fixation or open pedicle screw fixation (Fig. 4).

### Assessment of clinical outcomes

Co-morbidities were classified as cardiovascular, cerebrovascular, pulmonary, urologic, gastrointestinal, endocrinological, hepatobiliary, and history of cancer. Complications were classified as surgical approach-related complications and surgical approach-non-related complications. Clinical results were evaluated according to Numeric Rating Scale (NRS) for back and leg pain and Oswestry disability index (ODI) score. These scores were calculated before surgery and postoperatively at 3 months, 6 months, 1 year, 2 years, and the final follow-up visit. Patient satisfaction rate (PSR), return to daily activity, and surgical recommendation to others were evaluated at every follow-up visit. About the assessment of PSR, return to daily activity, and surgical recommendation to others was according to interview of patients at every follow-up periods.

### Assessment of radiological outcomes

**Fig. 5:**
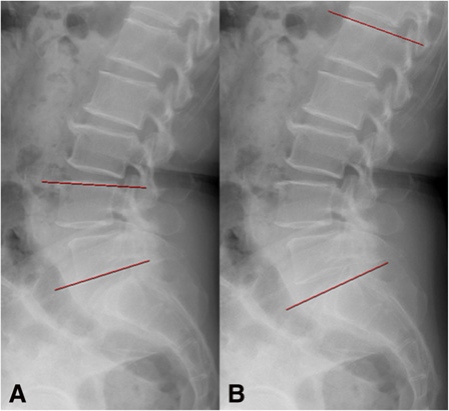
**a**. The segmental lordotic angle was measured between the upper endplate of the cranial side of the vertebral body and the lower endplate of the caudal side of the vertebral body for the operating level. **b**. The lumbar lordotic angle was measured between the upper endplate of the L1 vertebral body and the upper endplate of the S1 vertebral body

Routine conventional X-ray images were obtained before surgery, immediately after the operation, and at the final follow-up visit. Disc height was measured at the midpoint of the disc space on plain standing lateral radiography. The segmental lordotic angle was measured between the upper endplate of the cranial side of the vertebral body and the lower endplate of the caudal side of the vertebral body for the operating level (Fig. 5a) [15]. The lumbar lordotic angle was measured between the upper endplate of the L1 vertebral body and the upper endplate of the S1 vertebral body (Fig. 5b) [15]. Computed tomography (CT) images were obtained before surgery and postoperatively at 6 months, 1 year, 2 years, and at the final follow-up visit. Fusion status was analyzed using modified Bridwell fusion criteria [16, 17].

### Statistical analysis

Different parameters measured between the two age groups were assessed with *t* test for continuous variables and chi-square test or Fisher exact test for categorical variables. Data are presented as *n* (%) for categorical variables and mean ± standard deviation (SD) for continuous variables. All statistical analyses were performed using Statistical Analysis System (SAS) Version 9.4 (SAS Institute, Cary, NC, USA). Statistical significance was considered when the *p* value was less than 0.05.

## Results

### Patient characteristics

A total of 63 patients underwent OLIF procedure. The mean age of these patients was 67.1 years (range, 49–85 years). Demographic data of group A and group B are summarized in Table 1. There were statistically significant differences in age and bone mineral density (BMD) between the two groups. The mean hospital stay of all patients was 7.6 days (range, 3–24 days). Their mean operation time was 128.0 min (range, 55–325 min). Twelve patients (1 patient in group A, 6 patients in group B) needed the intraoperative transfusion. The mean blood transfusion volume was 29.8 ml (range, 0–400 ml). The mean follow-up duration was 20.4 months (range, 12–42 months). Preoperative diagnoses were degenerative spondylolisthesis (51.72% in group A and 52.94% in group B), lumbar stenosis (37.93% in group A and 38.24% in group B), and HNP (10.35% in group A and 8.82% in group B). Perioperative data of group A and group B are summarized in Table 2. However, based on our limited number of cases, even though there was no statistically significant difference in perioperative parameters between two groups, no difference might not guarantee the equal clinical outcomes between two groups.

**Table 1:** Demographics characteristics of patients under or over 65 years of age

	≤ 65 (group A)	> 65 (group B)	*p* value
Mean age: years (range)	60.1 ± 4.2 (49.0–65.0)	73.0 ± 4.6 (66.0–85.0)	< 0.0001^a^
Male/female: ratio	10:19	15:19	0.4359
Mean BMD: T-score (range)	− 1.2 ± 1.4 (− 3.8–1.5)	− 2.0 ± 1.6 (− 4.7–1.2)	0.0416^a^
BMD ≤ − 3.0: patients	3	13	0.0112^a^

*BMD* bone mineral density

^a^Statistical significance was considered when the *p* value was less than 0.05

**Table 2:** Perioperative data of patients under or over 65 years of age

	≤ 65 (group A)	> 65 (group B)	*p* value
Hospital stay: day (range)	6.8 ± 6.6 (3.0–20.0)	8.2 ± 8.4 (3.0–24.0)	0.1428
Anesthesia time: minute (range)	303.3 ± 102.2 (185.0–600.0)	337.2 ± 115.8 (170.0–710.0)	0.2262
Operation time: minutes (range)	122.0 ± 97.0 (60.0–250.0)	132.5 ± 113.8 (55.0–325.0)	0.3721
Intraoperative blood loss: ml (range)	253.4 ± 120.7 (50.0–700.0)	225.3 ± 120.7 (30.0–500.0)	0.6103
Blood transfusion: ml (range)	13.1 ± 7.8 (0.0–380.0)	44.1 ± 9.4 (0.0–400.0)	0.4179
Mean follow-up: months (range)	22.8 ± 11.2 (12.0–42.0)	18.5 ± 9.3 (12.0–37.0)	0.5134
Mean fusion level: number (range)	1.5 ± 0.8 (1–3)	1.5 ± 0.7 (1–3)	0.7988

### Clinical outcomes

**Fig. 6:**
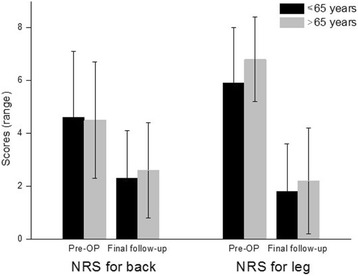
Changes of NRS score in group A and group B during the follow-up period

**Fig. 7:**
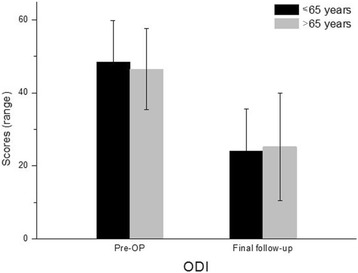
Changes of ODI score in group A and group B during the follow-up period

The most common co-morbidity was cardiovascular problem (38/63, 60.31%) such as hypertensive disorder (Table 3), followed by endocrinology disease (35/63, 55.56%). There were 17 patients with diabetes and 9 patients with rheumatoid arthritis. Results for the number of co-morbidities in the two age groups are summarized in Table 4. The mean NRS score for back pain was significantly decreased from 4.6 (range, 1.0–9.0) preoperatively to 2.3 (range, 0.0–7.0) at the final follow-up visit in group A. It was decreased from 4.5 (range, 0.0–8.0) preoperatively to 2.6 (range, 0.0–8.0) at the final follow-up visit in group B. The NRS score for leg pain was decreased from 5.9 (range, 2.0–10.0) preoperatively to 1.8 (range, 0.0–6.0) at the final follow-up visit in group A and from 6.8 (range, 2.0–9.0) preoperatively to 2.2 (range, 0.0–8.0) at the final follow-up visit in group B. The ODI score was decreased from 48.4% (range, 32.0–68.0%) preoperatively to 24.0% (range, 9.0–56.0%) at the final follow-up visit in group A and from 46.5% (range, 30.0–70.0%) preoperatively to 25.2% (range, 6.0–66.0%) at the final follow-up visit in group B. Detailed changes in NRS and ODI scores from preoperatively to the final follow-up visit of the two age groups are shown in Figs. 6 and 7. During the follow-up period, one patient in group A had an additional surgery due to the development of an adjacent segment disease. One patient in group B died during the follow-up period. The PSR, return to daily activity, and surgical recommendation to others in group A and group B are summarized in Table 5.

**Table 3:** Classification of co-morbidities

	Number of patients	Percentage
Cardiovascular	38	60.31
Endocrinologic	35	55.56
Pulmonary	4	6.35
Hepatobiliary	4	6.35
Cerebrovascular	3	4.76
Urologic	3	4.76
Gastrointestinal	1	1.59
History of cancer	4	6.34

**Table 4:** The number of co-morbidities in patients under or over 65 years of age

Number of co-morbidities in each patient	≤ 65 (group A)	> 65 (group B)	*p* value
0	8 (27.59%)	3 (8.82%)	0.0317^a^
1	9 (31.03%)	10 (29.41%)	0.8185
2	6 (20.69%)	13 (38.24%)	0.0483^a^
3	5 (17.24%)	4 (11.76%)	0.7389
4	1 (3.45%)	3 (8.82%)	0.0573
5	0 (0%)	1 (2.94%)	–

^a^Statistical significance was considered when the *p* value was less than 0.05

**Table 5:** PSR, return to daily activity, and surgical recommendation to others

	≤ 65 (group A)	> 65 (group B)	*p* value
PSR: score (range)	82.2 ± 15.1 (40.0–100.0)	79.6 ± 13.1 (50.0–100.0)	0.5205
Return to daily activity	92.00%	95.83%	1.0000
Surgical recommendation to others	88.00%	100.00%	0.2347

*PSR* patient’s satisfaction rate

### Radiological outcomes

The average fusion level of the 63 patients was 1.5 (range, 1–3 levels). Detailed results of fusion level in the two age groups are summarized in Tables 6 and 7. Based on modified Bridwell fusion criteria, grade A fusion rates at 1-year, 2-year, and the final follow-up visits in group A were 13, 80, and 63%, respectively, and grade B fusion rates were 80, 20, and 37%, respectively. In group B, grade A fusion rates at 1-year, 2-year, and the final follow-up visits were 37, 91, and 100%, respectively, while grade B fusion rates were 57, 9, and 0%, respectively (Table 8). Overall fusion rates at 1-year, 2-year, and the final follow-up visits were 93, 100, and 100% in group A, 94, 100, and 100% in group B.

**Table 6:** Results of fusion level

Number of level	≤ 65 (group A)	> 65 (group B)	*p* value
Total	44	50	0.4041
1 level	19	21	0.7518
2 levels	5	10	0.1967
3 levels	5	3	0.4795

**Table 7:** Results of fusion level site

Level site	≤ 65 (group A)	> 65 (group B)	*p* value
L2–3	6	9	0.4386
L3–4	12	23	0.0630
L4–5	25	18	0.2278

**Table 8:** Fusion rates during follow-up period

Follow-up period	Fusion grade	≤ 65 (group A)		> 65 (group B)		*p* value
1 year	Grade A	*n* = 27/29	13%	*n* = 32/34	37%	0.4812
	Grade B		80%		57%	
2 years	Grade A	*n* = 23/23	80%	*n* = 24/24	91%	0.4072
	Grade B		20%		9%	
> 2 years	Grade A	*n* = 7/7	63%	*n* = 2/2	100%	1.0000
	Grade B		37%		0%	

Disc height in group A was increased from 8.6 mm (range, 0.0–16.6 mm) preoperatively to 13.4 mm (range, 10.6–17.0 mm) postoperatively. At the final follow-up visit, disc height was decreased to 12.3 mm (range, 8.5–16.4 mm). Disc height in group B was increased from 8.9 mm (range, 2.9–17.4 mm) preoperatively to 13.9 mm (range, 9.0–18.9 mm) postoperatively. At the final follow-up visit, disc height was decreased to 12.9 mm (range, 5.6–16.2 mm) (Table 9). The segmental lordotic angle of the operating level in group A was increased from 10.1° (range, − 16.4°–25.1°) preoperatively to 15.2° (range, 1.5°–28.2°) postoperatively. At the final follow-up visit, the angle was decreased to 14.9° (range, 1.5°–28.0°). In group B, the segmental lordotic angle of the operating level was increased from 9.4° (range, − 0.3°–24.6°) preoperatively to 14.9° (range, 0.1°–27.8°) postoperatively. At the final follow-up visit, the angle was decreased to 14.2° (range, 0.8°–32.6°) (Table 9). The lumbar lordotic angle in group A was increased from 37.0° (range, 5.6°–56.5°) preoperatively to 42.5° (range, 24.3°–64.8°) postoperatively. At the final follow-up visit, the angle was increased to 41.9° (range, 25.3°–63.7°). The lumbar lordotic angle in group B was increased from 38.3° (range, 17.3°–59.9°) preoperatively to 43.8° (range, 24.4°–57.2°) postoperatively. At the final follow-up visit, the angle was decreased to 42.4° (range, 0.1°–57.9°) (Table 9).

**Table 9:** Changes in disc height, segmental lordotic angle, and lumbar lordotic angle

		≤ 65 (group A)	> 65 (group B)	*p* value
Disc height: mm (range)	Pre-OP	8.6 ± 2.9 (0.0–16.6)	8.9 ± 3.0 (2.9–14.7)	0.5327
	Post-OP	13.4 ± 1.8 (10.6–17.0)	13.9 ± 2.4 (9.0–18.9)	0.2196
	Final follow-up	12.3 ± 1.7 (8.5–16.4)	12.9 ± 2.1 (5.6–16.2)	0.6108
Segmental lordotic angle: degree (range)	Pre-OP	10.1 ± 7.8 (− 16.4–25.1)	9.4 ± 6.3 (−0.3–24.6)	0.6590
	Post-OP	15.2 ± 6.2 (1.5–28.2)	14.9 ± 6.0 (0.1–27.8)	0.0703
	Final follow-up	14.9 ± 6.4 (1.5–28.0)	14.2 ± 6.2 (0.8–32.6)	0.1645
Lumbar lordotic angle: degree (range)	Pre-OP	37.0 ± 12.2 (5.6–56.5)	38.3 ± 11.8 (17.3–59.9)	0.6633
	Post-OP	42.5 ± 9.9 (24.3–64.8)	43.8 ± 8.5 (24.4–57.2)	0.3159
	Final follow-up	41.9 ± 9.4 (25.3–63.7)	42.4 ± 13.1 (0.1–57.9)	0.3059

*OP* operation

### Complications

Overall perioperative complications occurred in 8 of 29 (27.6%) patients in group A and in 10 of 34 (29.4%) patients in group B. In group A, the isolated approach-related complication occurred in 8 of 29 (27.6%) patients while isolated approach-non-related complication occurred in 0 of 29 (0%) patients. In group B, the isolated approach-related complication occurred in 9 of 34 (26.5%) patients; isolated approach-non-related complication occurred in 1 of 34 (2.9%) patients. Statistical analysis results of complications of group A and group B are summarized in Table 10. Lumbar plexopathy was the most common approach-related complication (5 patients in group A, 6 patients in group B). All 11 patients were treated with conservative care, of which 10 gradually recovered within 3- to 6-month period while one patient had persistent left hip flexion weakness (grade 4+ at the final follow-up) but without any functional disability. Sympathetic chain symptoms were observed in 3 patients in group A and 1 patient in group B. Of the four patients, 3 gradually recovered within 2- to 4-month period. In the other one patient, symptoms persisted beyond 6 months. One patient had the intraoperative ventral dural tear which was treated with dural repair [18]. One patient had the intraoperative urethral injury which was treated with repair immediately by a urology surgeon [19]. The most common approach-non-related complication was postoperative respiratory complication. These were successfully treated with conservative treatment (Table 11). Fortunately, we do not have any fatal complications in these two groups enrolled.

**Table 10:** Complications in patients under or over 65 years of age

Complications	≤ 65 (group A)	> 65 (group B)	*p* value
Total	*n* = 8 (27.6%)	*n* = 10 (29.4%)	0.4339
Approach-related complication	*n* = 8 (27.6%)	*n* = 9 (26.5%)	0.7389
Approach-non-related complication	*n* = 0 (0%)	*n* = 1 (2.9%)	–

**Table 11:** Classification of complications

Complications	≤ 65 (group A)	> 65 (group B)
Approach-related complication		
Abdominal vascular injury	0	0
Ventral dural tear	0	1
Ureteral injury	0	1
Lumbar plexopathy (sensory)	5	4
Lumbar plexopathy (motor)	0	2
Sympathetic chain symptom	3	1
Screw malposition	0	0
Wound infection	0	0
Ileus	0	0
Approach-non-related complication		
Urinary system complication	0	0
Respiratory complication	0	1
Delirium	0	0

## Discussion

With increasing lifespan, the number of patients in need of fusion surgery is also increasing. High rates of postoperative morbidity and mortality have been reported [20, 21]. Based on reported in the literature, patients aged more than 65 years have higher rates of major complications [22, 23]. These increased rates of morbidity and mortality reflect that the number of systemic diseases and operative blood loss are increased and hospital stay is longer [24].

Traditional posterior lumbar interbody fusion can cause unavoidable damage to paraspinal back muscles, soft tissue, and posterior bone structure of the lumbar spine. Moreover, it can expose neural elements in the spinal canal to iatrogenic injury. In the last few decades, many surgeons have explored various surgical approaches, especially focusing on minimally invasive techniques, to achieve lumbar fusion while avoiding complications caused by posterior lumbar interbody fusion.

ALIF provides direct access to the intervertebral disc. It allows easy removal of disc and implant insertion with the excellent restoration of disc height and optimal correction of sagittal balance, thus reducing the incidence of ASD with relative preservation of spine anatomy [25–28]. However, ALIF can potentially cause vessel injury due to retraction [29]. An anterolateral and minimally invasive approach was introduced by Mayer in 1997 [3]. It was then modified by McAfee et al. [30] and Ozgur et al. [7] into a trans-muscular, psoas-splitting access, and extreme lateral trans-psoas approach for interbody fusion (XLIF). However, the XLIF approach has risks of causing neurologic injuries while dissecting the psoas muscle [31].

“Oblique lateral interbody fusion” or OLIF was introduced in 2012 [11]. This new approach reflects a paradigm shift in anterolateral spinal fusion surgery. It is a solution to the caveats of both ALIF and LLIF techniques [25, 32–35].

The largest cohort study up to date has described the outcome of the OLIF procedure [11]. From a cohort of 179 patients, 19 (10.6%) developed the single complication, while one individual had two complications. These complications were incisional pain (2.2%), sympathetic chain injury (1.7%), vascular injury to iliac and iliolumbar vessels (1.7%), neurological deficits (2 patients), and symptomatic L5–S1 pseudoarthrosis (1 patient), which were comparable to traditional approaches. Overall, this relatively large cohort study has demonstrated the feasibility of using anterolateral access for fusion of the lumbar spine. However, that study used a mini-open technique. Compared to that study, we demonstrated a further minimally invasive OLIF technique aided by tubular retractor system. Another study based on a nationwide survey in Japan [36] demonstrated that compared with extreme lateral trans-psoas interbody fusion, OLIF procedure can effectively reduce the incidence of related complications.

We included 63 patients in our study. The overall complication rate was 28.6%, which was 27.6% in patients under 65 years and 29.4% in those over 65 years of age. The most common approach-related complication was lumbar plexopathy (17.5%). Sensory lumbar plexopathy including left inguinal symptoms and left anterior thigh numbness was seen in 5 patients in group A and 4 patients in group B. All patients had transient symptoms. Motor lumbar plexopathy in the form of left hip flexion weakness was seen in 2 patients in group B. One patient was rapidly improved whereas the other patient had persistent weakness without functional debilitation at the final follow-up. Sympathetic chain symptoms were observed in 3 patients in group A and 1 patient in group B. Of the four patients, 3 gradually recovered within 2- to 4-month period. In the other one patient, symptoms persisted beyond 6 months. During the operation of OLIF, sympathetic chain symptoms could be injured because the position of sympathetic chain overlaps with OLIF corridor and surgeons need gentle dissection of the psoas muscles. But most complicated cases are transient and minor, and most of them improve themselves within 3 to 6 months after the operation. This complication was also reported after ALIF procedure [37]. In our study, the incidence of major complications, such as ureteral injury, vascular injury, and ventral dural damage, was only 3% (0% in group A, 3% in group B). No vascular injury was reported in both groups. In the elderly group, one patient reported intraoperative ventral dural damage [18] while another reported intraoperative ureteral injury [19]. In order to avoid any kind of injury on ventral dura mater, we recommend that the surgeon has to evaluate the proper OLIF angle before surgery and check intraoperative C-arm images frequently. To avoid the ureter injury, we also recommend using the tip of one’s finger to find the direction of the disc space and to dissect psoas muscle. In contrast to a surgical device, the fingertip is blunt and can feel the track of ureter. Although the complication rate of the elder group was slightly higher than that of the younger group, there was no significant difference in clinical outcome between the two groups based on clinical outcomes, patient’s satisfaction rate, and willingness to recommend it to others. However, based on our limited number of cases, even though there was no statistically significant difference in perioperative parameters between two groups, no difference might not guarantee the equal clinical outcomes between the two groups.

One of the major reasons for complications in spine fusion surgery is the higher frequency of co-morbidities in elderly patients. In our study, 82.5% (52/63) of patients had co-morbidities (72.4% in those under 65 years of age and 91.2% in those over 65 years of age). The most common co-morbidity was cardiovascular. Another major co-morbidity was diabetes. Many of these patients were on anti-platelets (or anti-coagulants) such as aspirin and clopidogrel. These drugs might increase the risk of perioperative bleeding. Osteoporosis is believed to be a risk factor for subsidence, screw loosening, and fusion failure [38–40]. In our study, the average T-score change on DEXA scan was − 1.2 in those under 65 years of age and − 1.9 in those over 65 years of age. During the perioperative period, 21% of patients who were under 65 years of age and 47% of patients of those who were over 65 years of age were treated with osteoporosis medication. In our study, one patient who was treated with conservative treatment had postoperative adjacent level lumbar vertebral compression fracture in group B. Subsidence occurred in 12 (27.3%) of 44 levels at 6 months and 1 year after surgery in group A. However, subsidence occurred in 19 (38%) of 50 levels at 6 months after surgery and 22 (40%) of 50 levels at 1 year after surgery in group B. There was no revision surgery related to subsidence of cage. Symptomatic pseudoarthrosis was not observed during the follow-up period in either group.

In our study, the segmental lordotic angle and the lumbar lordotic angle were increased immediately after the surgery. However, it was decreased in both groups as time went by. After a follow-up time of at least 1 year, significant clinical improvement in segmental lordotic angle and lumbar lordotic angle were observed in both group A and group B.

Our study had several limitations. First, it had a retrospective study design with a small sample size. In addition, the duration of follow-up was relatively short. Further follow-up is needed to strengthen these observations. In addition, a larger number of patients should be enrolled in future studies to confirm our findings.

## Conclusion

In this study, patients over 65 years of age had the slightly high incidence of complication compared with those younger than 65 years. However, based on clinical outcomes, patient’s satisfaction rate, and willingness to recommend it to others, OLIF in elderly patients also showed satisfactory clinical and radiological outcomes; OLIF may be considered as an alternative surgical method for degenerative spinal disease in the elder population.

## Abbreviations

ALIF: Anterior lumbar interbody fusion
ASD: Adjacent segment disease
BMD: Bone mineral density
CT: Computed tomography
DBM: Demineralized bone matrix
DDD: Degenerative disc disease
HNP: Herniated nucleus pulposus
IRB: Institutional review board
LLIF: Lateral lumbar interbody fusion
MIS: Minimally invasive spinal
NC: North Carolina
NRS: Numeric Rating Scale
ODI: Oswestry disability index
OLIF: Oblique lateral interbody fusion
OP: Operation
PLIF: Posterior lumbar interbody fusion
PSR: Patient’s satisfaction rate
SAS: Statistical Analysis System
SD: Standard deviation
TLIF: Transforaminal lumbar interbody fusion
USA: United States of America
XLIF: Extreme lateral trans-psoas approach for interbody fusion
