# Correction to: Outcomes of oblique lateral interbody fusion for degenerative lumbar disease in patients under or over 65 years of age

**DOI:** 10.1186/s13018-018-0766-5

**Published:** 2018-04-30

**Authors:** Chengzhen Jin, Milin S. Jaiswal, Sin-Soo Jeun, Kyeong-Sik Ryu, Jung-Woo Hur, Jin-Sung Kim

**Affiliations:** Spine Center, Department of Neurosurgery, Seoul St. Mary’s Hospital, College of Medicine, The Catholic University of Korea, Seoul, South Korea, 222 Banpo-daero Seocho-gu, Seoul, 06591 Republic of Korea

## Correction

The original publication of this article exhibits an error with respect to an introduced patient value found in the first paragraph of the Results section. The correct version can be found below.

Incorrect sentence:

Twelve patients (1 patient in group A, 6 patients in group B) needed the intraoperative transfusion.

Correct sentence:

Seven patients (1 patient in group A, 6 patients in group B) needed the intraoperative transfusion.
